# Supplementary material for: Ultrabroadband sound control with deep-subwavelength plasmacoustic metalayers
Source: Nat Commun. 2023 May 19;14:2874. doi: 10.1038/s41467-023-38522-5 (PMC10198984; doi:10.1038/s41467-023-38522-5)
Supplement: Supplementary file 1 — Supplementary Information [file 41467_2023_38522_MOESM1_ESM.pdf]

Supplementary material file for the paper "Ultrabroadband sound control with deep-subwavelength plasmacoustic metalayers"

Stanislav Sergeev[1], Romain Fleury[2], and Hervé Lissek[1]

[1] Signal Processing Laboratory LTS2, EPFL, Lausanne, Switzerland
[2] Laboratory of Wave Engineering, EPFL, Lausanne, Switzerland

Supplementary table 1. Physical and geometrical parameters of the discharge and air medium used to form the control transfer function $\theta(\omega)$.

Parameter	Symbol	Value	Unit
Air mass density	ρ_0	1.23	kg m^{-3}
Sound speed in air	c	343	m s^{-1}
Ambient temperature	T_0	293	K
Heat capacity	C_P	1015	J kg^{-1} K^{-1}
Inter-electrode gap	d	$6{\cdot}10^{-3}$	m
Discharge area	S	$25{\cdot}10^{-4}$	m^2
Microphone distance	x_0	$1 \cdot 10^{-2}$	m
Enclosure length	l	$25{\cdot}10^{-3}$	m
Load impedance	Z_l	$14.7{\cdot}10^3$	Pa s m^{-1}
Positive ion mobility	μ_i	$1.1{\cdot}10^{-4}$	m^2 V^{-1}s^{-1}
Voltage offset	U_{DC}	8.0	kV
Onset voltage	U_0	6.189	kV
Fitting constant	C	$2.2{\cdot}10^{-4}$	A V^{-2}

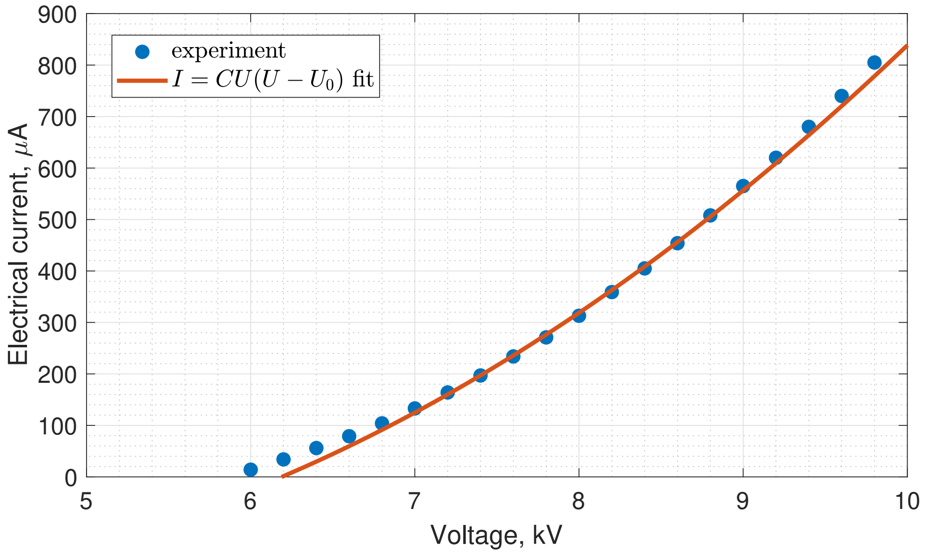

Supplementary figure 1. Measured voltage-current characteristics of the discharge and its fit with Townsend formula in the form $I = CU(U - U_0)$. Estimated parameters (with their 95% confidence bounds): C=2.20(2.13, 2.27) $\cdot 10^{-11}$ AV^{-2}, $U_0 = 6189\ (6123, 6255)$ V.

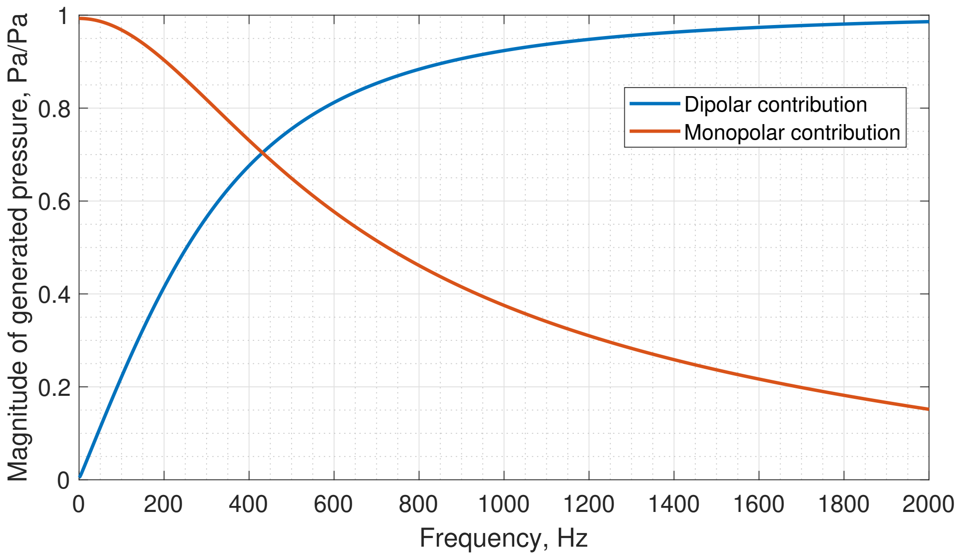

Supplementary figure 2. Magnitudes of sound pressure signals, derived from equation 7, generated by a plasmacoustic metalayer at the microphone position, in response to an incident sound source of amplitude 1 Pa.
